# Supplementary material for: A Pilot Study to Evaluate the Usefulness of Optical Coherence Tomography for Staging Iris Pigmented Lesions in Cats
Source: Vet Sci. 2024 Jun 7;11(6):261. doi: 10.3390/vetsci11060261 (PMC11209344; doi:10.3390/vetsci11060261)
Supplement: Supplementary file 1 [file vetsci-11-00261-s001.zip › vetsci-2948787-supplementary.pdf]

Supplementary Figure S1: Measurements and calculation of mean iris thickness and pigmented lesion to normal iris ratio.

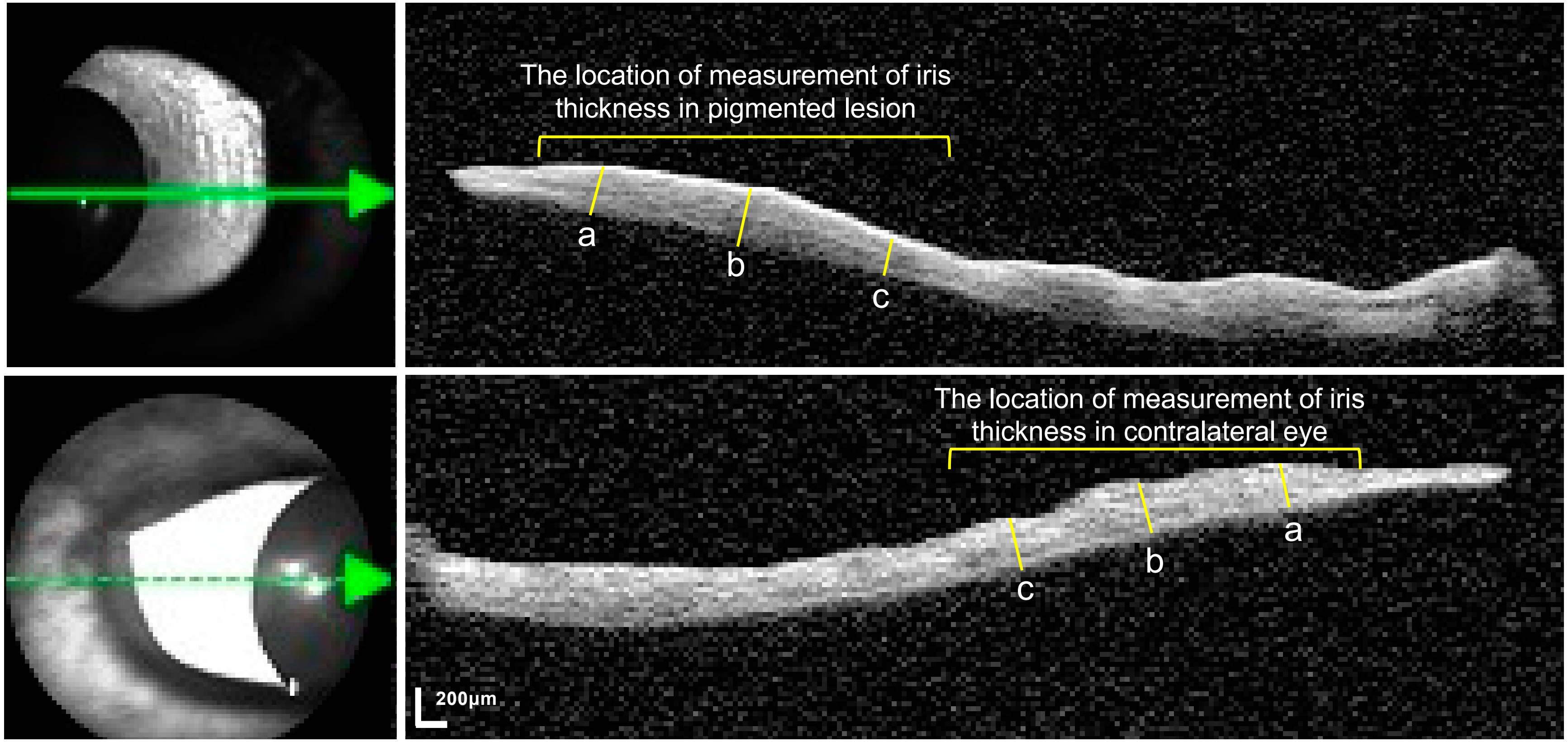

a; iris margin, b; center, c; iris root

Mean iris thickness (MIT) were calculated from the average of a + b + c.

Pigmented lesion to normal lesion ratio (PN ratio) were calculated from the division of MIT of pigmented lesion to normal lesion.

Supplementary Table S1: The list of measurements of the cases with suspected melanosis, iris melanosis, and early-FDIM.

Case No.	Diagnosis	Measurement location of pigmented lesion	The location of measurement of iris thickness in pigmented lesion (μm)			MIT in pigmented lesion (μm)	MIT in contralateral eye (μm)	PN ratio	Presence of hyperreflective line
			root	center	margin				
1	Iris melanosis	Root	228	219	222	223	201	1.11	Yes
2	Iris melanosis	Margin	438	477	438	451	480	0.94	Yes
3	Iris melanosis	Root	400	388	358	382	420	0.91	Yes
4	Early-FDIM	Root	484	529	445	486	379	1.28	No
5	Early-FDIM	Root	265	240	239	248	233	1.06	No
6	Early-FDIM	Root	423	451	406	427	284	1.50	No
7	Early-FDIM	Margin	266	246	246	253	191	1.33	No
SM1	Suspected melanosis	Root	366	402	431	400	390	1.03	No
SM2	Suspected melanosis	Margin	251	229	225	235	261	0.90	No
SM3	Suspected melanosis	Root	284	274	257	272	270	1.00	Yes
SM4	Suspected melanosis	Margin	150	129	120	133	130	1.02	Yes
SM5	Suspected melanosis	Root	510	470	433	471	392	1.20	Yes
SM6	Suspected melanosis	Root	431	388	377	399	349	1.14	Yes
SM7	Suspected melanosis	Margin	212	211	203	209	210	0.99	Yes
SM8	Suspected melanosis	Margin	215	204	199	206	239	0.86	Yes

MIT; mean iris thickness, PN; pigmented lesion to normal lesion

Supplementary Table S2: Intraclass correlation coefficient in MIT and PN ratio.

Intraobserver reproducibility				Interobserver reproducibility			
MIT		ICC(1,3)	95% CI	MIT		ICC(2,1)	95% CI
	Suspected melanosis	0.991	0.981 - 0.997		Suspected melanosis	0.952	0.866 - 0.983
	Iris melanosis	0.992	0.967 - 0.999		Iris melanosis	0.688	0.459 - 0.953
	early FDIM	0.997	0.989 - 0.999		early FDIM	0.762	0.12 - 0.950
	Total	0.996	0.992 - 0.998		Total	0.839	0.649 - 0.925
PN ratio				PN ratio			
	Suspected melanosis	0.837	0.475 - 0.964		Suspected melanosis	0.952	0.866 - 0.983
	Iris melanosis	0.836	0.331 - 0.953		Iris melanosis	0.972	0.030 - 0.999
	early FDIM	0.996	0.98 - 1.00		early FDIM	0.941	0.399 - 0.996
	Total	0.988	0.973 - 0.996		Total	0.896	0.667 - 0.996
